# Applications of Focused Ultrasound for the Treatment of Glioblastoma: A New Frontier

**DOI:** 10.3390/cancers14194920

**Published:** 2022-10-08

**Authors:** Andrew M. Hersh, Meghana Bhimreddy, Carly Weber-Levine, Kelly Jiang, Safwan Alomari, Nicholas Theodore, Amir Manbachi, Betty M. Tyler

**Affiliations:** 1 Department of Neurosurgery, Johns Hopkins University School of Medicine, Baltimore, MD 21287, USA; 2 Department of Biomedical Engineering, Johns Hopkins University School of Medicine, Baltimore, MD 21287, USA; 3 Department of Mechanical Engineering, Johns Hopkins University School of Medicine, Baltimore, MD 21287, USA; 4 Department of Electrical and Computer Engineering, Johns Hopkins University School of Medicine, Baltimore, MD 21287, USA; 5 Department of Anesthesiology and Critical Care Medicine, Johns Hopkins University School of Medicine, Baltimore, MD 21287, USA

**Keywords:** glioblastoma, focused ultrasound, FUS, targeted therapy, blood–brain barrier

## Abstract

**Simple Summary:**

Glioblastoma (GBM) is a common primary brain tumor with a short median overall survival despite aggressive treatment with resection, chemotherapy, and radiation therapy. Focused ultrasound (FUS) represents a promising new therapeutic approach for treatment of GBM. Unlike imaging forms of ultrasound, FUS can successfully penetrate the skull surrounding the brain, allowing for non-invasive ablation of tumor tissue. FUS can also temporarily disrupt the blood–brain barrier, a microvascular network that prohibits diffusion of most therapeutic agents, allowing chemotherapeutic drugs to penetrate the tumor. Other modalities are under investigation and include means of stimulating the immune system and sensitizing tumors to radiation therapy. The feasibility and safety of transcranial FUS has been illustrated in animal models and clinical trials. Precise results can be obtained under guidance from magnetic resonance imaging, limiting side effects. Successful outcomes from clinical trials will likely continue to motivate investigation and innovation of FUS technology for GBM.

**Abstract:**

Glioblastoma (GBM) is an aggressive primary astrocytoma associated with short overall survival. Treatment for GBM primarily consists of maximal safe surgical resection, radiation therapy, and chemotherapy using temozolomide. Nonetheless, recurrence and tumor progression is the norm, driven by tumor stem cell activity and a high mutational burden. Focused ultrasound (FUS) has shown promising results in preclinical and clinical trials for treatment of GBM and has received regulatory approval for the treatment of other neoplasms. Here, we review the range of applications for FUS in the treatment of GBM, which depend on parameters, including frequency, power, pulse duration, and duty cycle. Low-intensity FUS can be used to transiently open the blood–brain barrier (BBB), which restricts diffusion of most macromolecules and therapeutic agents into the brain. Under guidance from magnetic resonance imaging, the BBB can be targeted in a precise location to permit diffusion of molecules only at the vicinity of the tumor, preventing side effects to healthy tissue. BBB opening can also be used to improve detection of cell-free tumor DNA with liquid biopsies, allowing non-invasive diagnosis and identification of molecular mutations. High-intensity FUS can cause tumor ablation via a hyperthermic effect. Additionally, FUS can stimulate immunological attack of tumor cells, can activate sonosensitizers to exert cytotoxic effects on tumor tissue, and can sensitize tumors to radiation therapy. Finally, another mechanism under investigation, known as histotripsy, produces tumor ablation via acoustic cavitation rather than thermal effects.

## 1. Introduction

Glioblastoma (GBM) is the most frequent primary astrocytoma, representing over 15% of all adult brain tumors and 50% of all gliomas, and is also the most lethal [1]. GBM is also encountered in the pediatric population, although the frequency is smaller, estimated at 3–15% of primary tumors of the central nervous system (CNS) [2]. GBM is a fast-growing, invasive tumor with an overall survival rate around 14–15 months. The tumor is characterized by hemorrhage and necrosis and appears on imaging as an irregular lesion with a central area of necrosis surrounded by edema [3]. The mainstay of treatment consists of maximal safe surgical resection followed by adjuvant radiation therapy (RT) and chemotherapy using temozolomide (TMZ). Despite this aggressive regimen, the 5-year survival is only 10% [4]. Although gross total resection has been associated with improved outcomes, the feasibility of a maximal resection is affected by the risk of substantial neurological deficits and morbidity [5].

A high recurrence rate and resistance to chemotherapy and RT is driven by the mutational landscape and tumor stem cell activity of GBM. In addition, the impermeable nature of the blood-brain barrier (BBB) severely limits diffusion of therapeutic agents from the blood into the brain, posing a barrier for non-invasive treatment options [6,7,8]. Consequently, there is substantial interest in novel treatment options for GBM. Local drug therapy, such as implantation of wafers that release chemotherapeutics into the resection cavity, has been shown to improve survival [9,10]. Convection-enhanced delivery has also been examined and involves direct injection of therapeutics through an infusion catheter by generating a pressure gradient that drives fluid flow [11,12]. Non-invasive methods of bypassing the BBB have also been explored, including intranasal delivery, nanoparticle carriers, drug modification and viral vectors [13,14,15,16]. However, these non-invasive treatment options have not yet been validated clinically for routine practice.

Interest has arisen in focused ultrasound (FUS) to improve outcomes of patients with GBM. FUS technology can deliver beams of ultrasound to precise areas of the brain, targeting tumor cells and avoiding deleterious effects on healthy tissue. The range of applications for FUS technology are broad, including high-intensity thermal ablation of tumor tissue and low-intensity transient opening of the BBB to improve therapeutic delivery to tumor tissue [17,18]. In addition, FUS is being explored for its capacity to stimulate the immune system to attack tumor cells and to sensitize GBM to RT [19,20]. Presently, FUS technology is approved by the United States Food and Drug Administration for treatment of several cancers, including prostate cancer, uterine leiomyoma, and bone metastases, and is under investigation for other neoplasms [21,22,23]. Approved applications for CNS pathology include treatment of essential tremor and Parkinson’s disease. FUS is also under exploration for treatment of Alzheimer’s disease, epilepsy, neuropathic pain, and its role in neuromodulatory therapies is under investigation [24,25].

The approval of FUS technology for treatment of neoplasms and intracranial pathology has further stimulated interest in its role for patients with GBM. In vitro and in vivo animal research has established the capacity of FUS to improve survival and reduce growth of GBM tissue. Clinical trials exploring FUS in patients with GBM are ongoing. Here, we review the principles of FUS technology and discuss applications of FUS under investigation for GBM.

## 2. Overview of Focused Ultrasound

### 2.1. High and Low Intensity Focused Ultrasound

Ultrasonic waves propagate with a frequency above the range of human hearing (>20 kHz) and are used extensively for diagnostic imaging and therapeutic purposes. Ultrasound technology is advantageous over other imaging modalities given its capacity to offer real-time, non-invasive imaging at a low cost. FUS is an ultrasound modality that typically uses a concave transducer to converge sound waves into a focused beam [26]. In 1942, Lynn et al. described the first study of FUS for intracerebral ablation in animals [27]. Advancements in FUS technology in recent years have improved its feasibility and safety for treatment of intracranial pathology, and it is being researched for treatment applications in a broad array of conditions including GBM, Parkinson’s disease, essential tremor, neuropathic pain, and thrombolysis [28]. 

FUS is categorized as high intensity FUS (HIFUS) or low intensity FUS (LIFUS). HIFUS intensities range between 100–10,000 W/cm^2^, whereas LIFUS has intensities in the 0.125–3 W/cm^2^ range [29]. The ultrasonic waves can be focused in several different ways. The simplest way is through the spherical curvature of the transducer, with the beam focused at a position determined by the transducer specifications. Alternatively, an interchangeable acoustic lens system can enable different properties, including focal length and geometry. A common mechanism of focusing the waves uses a phased-array transducer. The ”phased” component refers to the timing of the ultrasound beams, while the term ”array’ references the numerous individual elements, or transducers, that can each be pulsed independently.The elements are placed in a row and the timing of wave transmission varies throughout the array and is controlled so that the waves arise in phase at a target location. The computer integrates the data from the individual beams into an ultrasound image. The phased-array is the most versatile and most efficient technique designed for focusing ultrasound [30]. 

**Figure 1:**
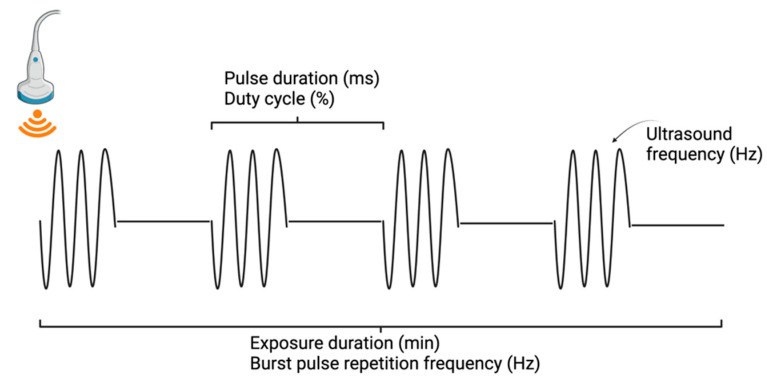
Depiction of ultrasound parameters. Created with BioRender.com.

There are several parameters to consider in ultrasound applications, including acoustic pressure (MPa), pulse duration (milliseconds), and the fundamental frequency (Hz), representing the number of wave cycles per second (Figure 1). Higher frequencies, particularly those above 1 MHz, improve the resolution of the image but come at the cost of decreased depth penetration. Most FUS-related studies employ probes with frequencies in the 200–600 kHz range. The duty cycle (%) is defined as the proportion of each pulse that is propagating ultrasound waves—that is, the proportion of time during which ultrasound waves are generated. The exposure duration (minutes) is the total time that tissue is targeted with ultrasound, which in turn determines the total intensity. Next, the pulse repetition frequency measures how many pulses occur each second [26]. Finally, the mechanical index measures the bio-effects of the ultrasound transducer and is directly proportional to the peak negative pressure and inversely proportional to frequency [31]. Choosing the appropriate ultrasound parameters is essential for achieving the desired biologic effect, and parameter modulation can be used to produce different outcomes. 

### 2.2. Mechanisms of Focused Ultrasound

Traditionally, intracranial ultrasound has been limited by an inability to penetrate the thick skull bones surrounding the brain, necessitating a craniectomy for its application to intracranial pathology. As ultrasound passes through bone, the irregularities and variations in thickness of the skull leads to attenuation, reflection, and wave distortion, adversely affecting the feasibility of ultrasound imaging and treatment [29]. However, improvements in the technology and mathematical calculations that can account for bony distortions and irregularities have rendered transcranial FUS feasible. Transcranial focusing generally uses a hemispherical phased-array transducer with an external cooling system to help avoid thermal injury [28]. 

The mechanisms behind FUS can be broadly divided between thermal and nonthermal effects. HIFUS exerts a thermal effect, heating the tissue through which it passes by causing vibration of molecules. The hyperthermia results in protein denaturation and DNA fragmentation. The absorbed energy from HIFUS can quickly raise the temperature above 60 °C, producing thermo-ablation and coagulation necrosis [29]. Indeed, a temperature greater than 56 °C maintained for just 2 s has been shown to cause coagulative necrosis [28,32]. LIFUS, in contrast, employs predominantly nonthermal effects, relying on mechanical perturbation and acoustic cavitation. Cavitation refers to the oscillation and collapse of gas bubbles in response to the compression and refraction of the ultrasonic pressure wave, with the effect of cavitation dependent on the pressure, frequency, and presence of gas in the medium. LIFUS is therefore generally used in conjunction with microbubbles, which can be delivered intravenously and travel to the site targeted by the transducer [33]. Stable cavitation is traditionally applied in FUS-mediated applications for intracranial tumors, which improves membrane permeabilty of the BBB and loosens tight junctions to support therapeutic drug delivery. In contrast, inertial cavitation produces direct mechanical damage [19]. Treatment of GBM using HIFUS would primarily be directed at thermal ablation of tumor tissue and the surrounding tumor microenvironment, while LIFUS can be used to improve therapeutic drug delivery and aid liquid biopsies. 

### 2.3. Focused Ultrasound Technology

Several commercial FUS systems are currently available for intracranial research. In 2016, the FDA approved the first FUS system, the ExAblate Neuro (InSightec, Dallas, TX, USA), for the treatment of essential tremor [34]. Its approval was later expanded in 2021 to also include Parkinson’s disease. The ExAblate is a transcranial magnetic resonance-guided FUS (MRgFUS), operating at a frequency of 650 KHz with pulses from 5–60 s durations, and it is generally for ablation procedures [35]. NaviFUS (NaviFUS, Taipei City, Taiwan) is a neuronavigation-guided FUS and is aligned using a patient’s computed tomography scan rather than magnetic resonance imaging. This device is used predominantly for treatment of neoplasms by disrupting the BBB to improve therapeutic drug delivery and for epilepsy using neuromodulation. The technology relies on cavitation effects from microbubbles to improve drug delivery. NaviFUS completed a clinical trial for GBM treatment in 2019 in Taiwan and received approval to be studied in a GBM clinical trial in the United States [36]. Finally, SonoCloud (Carthera, Paris, France) is a device implanted beneath the skin in a skull window, rather than relying on external transcranial FUS. The device uses LIFUS to stimulate microbubbles and disrupt the BBB to improve drug delivery, with a primary focus on improving treatment for patients with Alzheimer’s disease [37]. 

## 3. Enhanced Drug Delivery

The BBB poses a major obstacle to effective delivery of chemotherapeutics to GBM tissue. The BBB microvascular network consists of specialized tight junctions and adherens junctions connecting endothelial cells and restricting movement across the barrier primarily to small nonpolar compounds [13,38,39,40]. Furthermore, drugs that can cross the BBB often fail to accumulate in sufficient concentrations at target tissue due to multidrug efflux transporters that actively remove compounds from the intracranial space [41]. The chemotherapeutic agent TMZ is unique in its ability to cross the BBB, owing to its small size and lipophilic nature, although its concentration in brain tissue is smaller compared to its serum concentration, and its use results in systemic toxicity [42,43]. However, the BBB restricts transport of nearly all large chemotherapeutics and the vast majority of small therapeutic agents [14,44]. GBM treated with TMZ commonly recurs, in part due to resistance mechanisms mediated by GBM stem cells that regenerate the tumor population depleted by chemotherapy [45]. Given the impermeability of the BBB to most therapeutic agents, a commonly used treatment strategy that improves survival time consists of polymer implants placed directly at the tumor site following surgical resection which release chemotherapeutics, such as carmustine, to the local tumor vicinity [9,10].

### 3.1. FUS-Mediated Opening of the BBB

There remains a critical need for noninvasive methods of therapeutic delivery across the BBB to further improve outcomes and survival in patients with GBM. FUS has been explored as a method of transiently and non-invasively increasing the permeability of the BBB to enhance therapeutic delivery. BBB opening is achieved through pulsed waves traveling at low frequencies (around 500 kHz) and low-pressure amplitudes which excite ultrasound contrast agents or microbubbles injected intravenously. These particles oscillate in the presence of the ultrasound wave, expanding and contracting to produce a stable cavitation effect that disrupts the tight junctions of the endothelial cells [44]. Higher transducer frequencies are used to improve focal targeting [46]. The effect is transient, lasting around 4–6 h, limiting side effects by ensuring that the impermeable state of the BBB is quickly restored to prevent permanent diffusion of toxic compounds [44]. 

**Figure 2:**
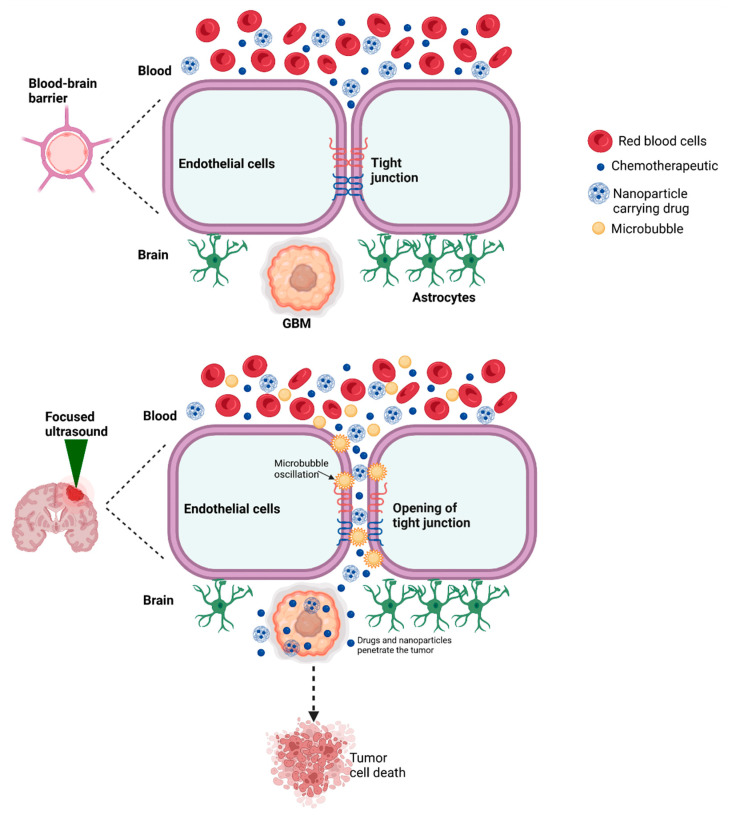
The BBB is a microvascular network that restricts diffusion of most molecules across the brain. The barrier includes a series of tight junctions, including occludins and claudin molecules. Application of FUS stimulates oscillations of microbubbles, producing acoustic cavitation that interferes with tight junction interactions and transiently opens the BBB. Nanoparticles and therapeutic agents can diffuse across the BBB to target the tumor. The BBB is opened only in the region stimulated by FUS, ensuring precise delivery of therapeutics to tumor tissue and limiting side effects to healthy tissue.

The precise cellular mechanisms underlying these FUS-induced changes are unknown and remain an active area of investigation [47]. The oscillating microbubbles stretch the walls of the vascular BBB, promoting deformation of the cellular membrane and activating mechanosensitive ion channels in the endothelial cells of the BBB that result in increased permeability (Figure 2). Cellular changes have been observed after FUS, including an increase in cytoplasmic channels and vesicles, along with a reduction in tight junction and gap junction proteins, including claudins, occludins, and zonula occludens that connect membrane proteins with the actin cytoskeleton [48]. The interactions between these proteins is also altered, and FUS is believed to promote changes in key signaling pathways, such as the phosphatidylinositol 3-kinase/Akt signaling pathway that in turn regulates permeability of the BBB [48]. Furthermore, FUS may inhibit the action of multidrug efflux transporters, preventing the removal of therapeutics [49]. These cellular changes allow for transcellular and paracellular pathways across the BBB [46]. FUS can also modify the extracellular space using radiation force-induced displacements to increase the interstitial space and improve dispersion of NPs [50,51].

The microbubbles are a critical component of FUS-mediated drug delivery, reducing the prerequisite ultrasound power 100-fold by concentrating the energy generated by the ultrasound waves, preventing deleterious damage to brain tissue and overheating of the skull [46]. Microbubbles range between 1–10 µm in diameter, while nanobubbles on the scale of 100 nm–1 µm are gaining popularity due to their smaller size, which improves their ability to travel through tumor vascular pores and reduces their cavitation. Several ultrasound contrast agents are clinically approved for BBB disruption, and development of new agents should consider the influence of size and chemical properties on the bubble’s stability during storage, stability during in vivo circulation, and cavitation threshold [52]. 

The precision of FUS for treatment of intracranial GBM can be enhanced using MRI, ensuring disruption of cancerous tissue while minimizing the effects on healthy tissue [44]. MRgFUS can also be used for real-time monitoring of the biological effects of FUS in conjunction with acoustic monitoring of microbubble emissions. Furthermore, the opening of the BBB can be confirmed with contrast-enhanced MRI, which demonstrates focal extravasation of contrast at the target tissue [49]. 

### 3.2. Applications of FUS for Drug Delivery

LIFUS-mediated BBB opening has been used to deliver TMZ to brain tumors, as well as various chemotherapeutic agents that traditionally exhibit low permeability across the BBB. For example, Wei et al. illustrated that FUS-mediated BBB opening could promote accumulation of TMZ in brain tissue and extend median survival, although the effect was only for an additional 3 days [53]. Liu et al. also showed improved delivery of TMZ to the brains of nude mice implanted with glioma cells, conferring a survival benefit that improved substantially compared to controls as the dose of TMZ was increased [54]. Separately, Wei et al. used an orthotopic murine glioma model to evaluate FUS-mediated BBB opening for the delivery of etoposide to tumor tissue, which traditionally is rendered ineffective by the impermeability of the BBB. MRI confirmed the success of BBB opening while liquid chromatography-mass spectrometry showed an increased concentration of etoposide in brain tissue nearly 8-fold compared to mice treated without ultrasound. Most significantly, the treatment decreased tumor growth by 45% and improved median survival by 30% [55]. 

MRgFUS can also be used to improve permeability of nanoparticles across the BBB, which can be loaded with therapeutic agents, imaging agents, or both [56]. Nanoparticles are versatile small molecules designed for crossing the BBB, and include polymers, liposomes, dendrimers, metals, and nanogels [13]. They can be enhanced with surface ligands that promote site-specific targeting to particular brain regions, increase circulation time, and target BBB cellular receptors to improve permeability [57,58,59,60]. The nanoparticles can also be loaded with chemotherapeutic agents, allowing these nanoparticles to effectively function as “Trojan horses” carrying anti-tumor compounds to the target tissue [13]. For example, an orthotopic mouse model of GBM showed that MRgFUS could improve penetration of both the chemotherapeutic agent cisplatin and gold nanoparticles carrying cisplatin to brain tumors, which may improve the treatment effect from cisplatin [61]. The delivery efficiency of liposomal nanoparticles carrying the antineoplastic agent paclitaxel was improved in the setting of pulsed LIFUS, extending survival time of nude mice with GBM by 20% compared to control mice [62]. Separately, a study by Timbie et al. using a rat glioma model showed that MRgFUS could improve permeability of a biodegradable polymeric nanoparticle conjugated with cisplatin and chains of polyethylene glycol which increase circulation time of the nanoparticle. A corresponding reduction in tumor invasiveness and growth was noted with the treatment, further highlighting the potential clinical value of MRgFUS for nanoparticle-mediated treatment of GBM [63]. 

In addition to therapeutic applications, FUS treatment can also be performed to improve the permeability of nanoparticles carrying imaging agents across the BBB, allowing for the enhanced monitoring of the tumor and improving the precision of therapeutic delivery. For example, Chan et al. designed iron-platinum nanoparticles which improve the resolution of T2-weighted MRI and whose magnetic properties can be exploited to guide the particle to the tumor location. The chemotherapeutic doxorubicin was loaded to these nanoparticles, providing a single platform for imaging and treatment in tandem with FUS for BBB permeability [64].

Clinical trials examining the effects of MRgFUS for BBB opening are underway, and several experiments have shown promising results. A Phase 0 clinical trial of four patients illustrated that BBB opening can be performed noninvasively in a safe and tightly controlled manner [65]. A small clinical trial of six patients with GBM treated with MRgFUS who underwent multiple cycles of BBB disruption to improve penetration of TMZ chemotherapy showed that MRgFUS is safe without long-term complications at 1-year follow-up. Longer-term studies are needed to assess survival benefits, but all subjects survived past 1 year, while tumor recurrence was noted in two patients at 11 and 16 months [42]. Notably, GBM recurrence usually occurs after only 7 months, suggesting a benefit from FUS-mediated BBB opening, although larger sample sizes are needed [42]. 

An alternative approach adopted by Carpentier et al. consisted of designing a pulsed ultrasound device, the SonoCloud, that is implanted directly into patients’ skulls. A Phase 1/2a trial showed that the device could disrupt the BBB following the injection of microbubbles during monthly treatment sessions of carboplatin chemotherapy. MRI and clinical examination findings did not reveal concerns for side effects [66]. Later, a single-arm trial of 21 patients using the implanted device prior to intravenous carboplatin showed improved overall survival of 13 months in patients with clear BBB disruption on MRI compared to survival of 8.6 months in patients with poor disruption. Transient edema was noted in some cases, but no concerning adverse effects occurred [67].

## 4. Tumor Ablation

FUS technology can achieve direct ablation of tumor tissue by hyperthermia generated from the continuous exposure of tissue to high rates of energy deposition. Coagulative necrosis and protein denaturation result in tumor cell death at temperatures above 55 °C [68]. Traditionally, a craniectomy is required to remove the bone overlying the brain tissue to prevent attenuation of the HIFUS waves by the skull as well as adverse effects from overheating of the bone. Consequently, the approach is generally only performed in conjunction with surgical management. A Phase I clinical study using MRgFUS for precise ablation of brain tumors was performed in three patients 7–10 days after craniectomy, resulting in immediate changes on MRI and histological findings of thermocoagulation. However, early studies were limited by older software that occasionally resulted in ablation of tissue outside the target area [69]. 

A potential non-invasive approach for tumor thermocoagulation was illustrated by McDannold et al., who also used MR temperature imaging to visualize the degree of hyperthermia in three patients treated with HIFUS. However, the study was limited by the available device’s power which was insufficient to achieve thermal coagulation, as well as a narrow safety profile with sonication-related pain occurring in one patient [70]. Coluccia extended this work by highlighting a case of a 63-year-old patient treated with 25 high-power MRgFUS sonications using the ExAblate Neuro system at 650 kHz with pulses of 10–25 s durations. Partial tumor ablation was achieved, and no adverse effects occurred, but the study sample was too small to generalize to the broader population [71]. Recently, MacDonell et al. proposed an interstitial HIFUS device using an intraparenchymal catheter to deliver hyperthermia directly at the tumor tissue, avoiding attenuation from the skull and improving treatment margins [18]. Animal studies have demonstrated feasibility, but its clinical success has not yet been validated.

Additionally, hyperthermia may sensitize glioma stem cells to radiation, an effect believed to be mediated in part from hyperthermic inhibition of DNA repair, activation of innate and adaptive immune systems, and downregulation of hypoxia [72]. Hyperthermia also sensitizes glioma stem cells to radiation therapy by downregulating the Akt signaling pathway, a key mediator of stemness and self-renewal [73]. Consequently, there may be a role for HIFUS in improving tumor responsiveness to RT, but studies are needed to determine its role. 

Presently, FUS-mediated hyperthermia for treatment of GBM plays a limited role in GBM treatment. Limitations of the above studies include attenuation from the calvarium, ablation of healthy tissue in the path of the ultrasound waves, and technological limitations. Nonetheless, tumor ablation using HIFUS is used clinically for treatment of pancreatic, breast, and prostate tumors, along with uterine fibroids [74]. Additional studies examining the safety, feasibility, and efficacy of FUS-mediated hyperthermia is needed to consider its use in GBM.

## 5. Immunotherapy

Though chemotherapy is often very effective against tumor cells, systemic toxicity poses a major restriction on the dosing of therapeutic agents. Immunotherapy is another treatment option that exploits the body’s natural immune system to recognize tumor antigens and attack tumor cells, rendered possible in part by the high burden of mutations present in tumor cells. Examples include cancer vaccines containing tumor antigens that trigger a host T-cell response, chimeric antigen receptor-T cell therapy that genetically modifies host T cells to selectively target cancer cells, and immune checkpoint inhibitors that disrupt mechanisms by which tumor cells prevent recognition by the immune system [75,76]. FUS has been used to promote the delivery of immunotherapy drugs otherwise blocked by the impermeable nature of the BBB, including programmed death protein-1 and interleukin-12 [77,78]. 

The administration of immunotherapy drugs in the presence of FUS has been shown in animal models to significantly increase delivery to the brain, impair tumor growth, and prolong survival in animal models. Anti-programmed cell death-ligand 1 antibody has been shown to target cancer cells by preventing the binding of the PD-L1 protein on cancer cells with the PD-1 receptor on T cells, which normally results in the silencing of the T cell response. FUS has been shown to promote the targeting of the antibody to tumor cells [79]. Separately, Chen et al. administered IL-12 intraperitoneally during FUS treatment of a glioma rat model, finding that the combination of FUS and IL-12 administration exerted a significant effect on cytotoxic T lymphocyte population in the tumor environment compared to IL-12 administration alone. Tumor growth was suppressed as a result of treatment [77]. Sheybani et al. investigated effectiveness of FUS for administration of an antibody that targets CD47, a protein overexpressed on cancer cells which signals macrophages to inhibit phagocytosis. They found that timing the antibody after FUS disruption significantly improves delivery to gliomas, and the antibody administration improved survival in mouse models [80].

The mechanical perturbations induced by FUS, in addition to improving BBB permeability to immunomodulatory agents, can produce a transient inflammatory effect that favors tumor targeting. The FUS stimulation of microbubbles improves innate and adaptive immunity and activates T cells in the traditionally immunosuppressive tumor microenvironment [81,82]. FUS-mediated hyperthermia also increases extracellular vesicle release from glioma cells and modulates their proteomic profile, causing a decrease in markers of tumor growth and increase in pro-inflammatory markers that upregulate activity of dendritic cells [83]. 

## 6. Sensitization Strategies: Sonodynamic Therapy

Sonodynamic therapy (SDT) is an emerging modality that features the use of an ultrasound transducer to activate special chemical compounds known as sonosensitizers that accumulate in tumors, resulting in the release of reactive oxygen species (ROS) and cavitation bubbles upon sonication [84]. The technology builds upon photodynamic therapy (PDT), in which photosensitizers are irradiated and activated by an external light source with a wavelength matching their absorption spectrum [85,86]. Activation produces cytotoxicity that induces formation of ROS, deactivates signal transduction pathways, increases hypoxia and neo-angiogenesis, and induces necrosis and apoptosis [87,88,89,90,91]. Although PDT can selectively target tumors, its penetration depth is limited to approximately 1.5–2.4 mm at wavelengths of 600–850 nm, limiting its use for deep lesions [92]. In contrast, typical frequencies for SDT range above 20 kHz, allowing for the application of a narrow and focused ultrasound beam to a depth of nearly 7–10 cm [93,94]. 

Similarly to PDT, the cytotoxic effects of SDT involve ROS generation and, to a lesser extent, ultrasonic cavitation from microbubble oscillation [74,95]. Nonlinear low-pressure oscillation results in stable cavitation, as seen in LIFUS-mediated BBB opening, whereas violent oscillations with rapid growth and collapse lead to inertial cavitation [96]. The latter form of cavitation leads to thermal production and mechanical shearing of tumor cells [97,98]. ROS, including peroxide, superoxide, and hydroxyl radical, result from the release of energy upon excitation of sonosensitizers from their ground state [99]. The cytotoxic effects of ROS can injure mitochondrial membranes, contribute to cellular swelling, elevate cytoplasmic calcium levels, and cause lipid peroxidation. The consequences of severe damage from ROS results in cellular apoptosis [100,101,102,103]. 

SDT also exhibits less well-understood immunomodulatory mechanisms, such as a shift from anti-inflammatory M2 macrophages to pro-inflammatory M1 macrophages [104]. These M1 macrophages contribute to tumor death [105]. SDT may also function to promote maturation of dendritic cells in the tumor microenvironment as evidenced by higher levels of CD68 and CD80 in SDT-treated mice [106]. 

### 6.1. Sonosensitizers

Conventional sonosensitizers have been developed based on different types of molecules and include 5-Aminolevulinic acid (5-ALA), porphyrin derivatives, phthalocyanines, xanthenes, anti-tumor agents (e.g., adriamycin, artemisinin), anti-inflammatory drugs (e.g., piroxicam), inorganic sonosensitizers, and hypocrellin [96,107]. The composition of the sonosensitizer influences its physical and chemical properties and clinical applications. For example, inorganic sonosensitizers exhibit higher chemical and physiological stability compared to other compounds [108]. 5-ALA has a high safety profile and is already used clinically to improve rates of tumor resection by causing fluorescence of glioma, permitting fluorescence-guided surgery [109,110].

SDT for glioma treatment has been studied using both small animal intracranial and subcutaneous glioma xenograft models (Table 1). In these studies, the most common sonosensitizers tested include 5-ALA, sinoporphyrin, fluorescein, and hematoporphyrin monomethyl ether [85,100,111,112,113,114]. Studies show inhibition of intracranial and subcutaneous glioma growth, as well as increased survival for animals treated with SDT as compared to controls or animals receiving FUS without sonosensitizers [115,116,117,118]. 

Nanoplatforms are being developed to improve BBB penetration, increase target accumulation, and incorporate MRI capabilities to SDT [95]. The nanocarrier can improve permeability across the BBB and can be modified with targeting compounds to ensure precise tumor localization [140,141]. For example, SDT using sinoporphyrin sodium as the sonosensitizer encapsulated into nanoliposomes showed greater efficiency inhibiting glioma growth in orthotopically implanted mice than controls that received only saline or sinoporphyrin sodium [116,121]. Inorganic sonosensitizers, such as manganese ion chelating nanoassemblies, can serve as contrast agents for MRI to visualize nanoparticle accumulation and monitor treatment response. These sonosensitizers have shown inhibitory effects on glioma progression [94]. Protoporphyrin loaded in manganese dioxide nanocrystals can target the transferrin receptor to cross the BBB and selectively accumulate at the tumor site [135]. Protoporphyrin acts as a conventional sonosensitizer to damage tumor cells upon application of SDT, while the acidic tumor microenvironment facilitates the release of Mn^2+^ which damages the mitochondria and also results in production of ROS [142]. A biodegradable nanoplatform was recently developed that encapsulates catalase into silica nanoparticles containing the sonosensitizer indocyanine green. The nanoplatform has a long circulation time and can self-supply oxygen, while glutathione within the tumor cells promotes release of catalase from the nanoplatform, catalyzing formation of hydrogen peroxide and improving SDT efficiency [130]. 

### 6.2. Sonodynamic Therapy: Preclinical and Clinical Trials

Promising results have been found using SDT for treatment of GBM. The investigation of optimal FUS parameters for SDT in a rat glioma model indicated that acoustic power of 25 W/cm^2^ at 1 MHz for 5 min caused optimal selective antitumor effects [143]. The safety of SDT has also been investigated in experimental studies with large animal models. LIFUS with fluorescein and 5-ALA performed in eight healthy swine brains under MRI guidance did not produce necrosis nor apoptosis [144]. Rats implanted with glioma cells intracranially treated with SDT using the sonosensitizer 5-ALA showed significantly reduced tumor sizes compared to non-treated controls [145]. Chen et al. tested a novel sonosensitizer, ZnPcS2P2, on human glioma cells and showed the successful inhibition of tumor growth rate by inducing apoptosis and necrosis, evident by increased expression of caspase-3, caspase-8, and caspase-9 [146]. Prada et al. tested the cytotoxicity of SDT using fluorescein in a rat glioma model and found significant inhibition of ectopic malignant gliomas with selective accumulation in tumor areas where the BBB was disrupted by LIFUS [123]. Xu et al. showed that SDT combined with the photosensitizer Photofrin had improved antitumor activity when pretreated with fumitremorgin C, an inhibitor of ATP binding cassette subfamily G member 2 that normally acts to remove Photofrin from tumor cells [147]. Consequently, fumitremorgin C may play a beneficial role in SDT. SDT therapy for GBM has also been suggested to cause downregulation of multidrug resistance proteins, which can improve penetration of therapeutic agents to tumors [136]. 

The synergistic effects of SDT in combination with other therapies often improves efficacy of treatment. Many photosensitizers are also sonosensitizers, allowing for the combination of SDT and PDT to enhance tumor ablation effects. SDT with the photosensitizer Photochlor can promote enhanced tumor degradation in a mouse model of glioma [148]. Indeed, the combination of SDT and PDT results in greater decreases in tumor growth than either treatment alone [138,149]. SDT also activates the mitochondrial caspase apoptosis pathway, improving the sensitivity of tumor cells to chemotherapeutic agents by upregulating apoptosis proteins such as Bax, cleaved caspase-3, and cytochrome c [95,103]. Furthermore, SDT and PDT have been tested in combination with chemotherapeutics, such as bleomycin, which significantly inhibited the capacity of glioma cells to form clonogenic colonies and self-renew [150]. Similarly, SDT applied with nanoparticles containing the chemotherapeutic paclitaxel resulted in tumor inhibition and apoptosis in human glioma U87 cells and U87 tumor-bearing mice, further confirming the benefits of sonochemotherapy [132]. 

The clinical trials of SDT therapy have been limited for GBM, focusing instead on other solid tumors such as breast cancer and lung adenocarcinoma [151,152]. Still, several clinical trials investigating the use of 5-ALA with FUS for SDT are underway or being prepared in the GBM population. 5-ALA is a preferred sonosensitizer given that it is already clinically used for intraoperative fluorescence-guided glioma surgery [109,110]. One study, a phase 0 single center trial of 30 participants with recurrent high-grade gliomas, features intravenous administration of 5-ALA 6–7 h prior to MRgFUS for application of SDT (NCT04559685). Similarly, a Phase 1 multi-center study is underway investigating 5-ALA in recurrent high grade glioma (NCT05362409). Additionally, a Phase 1/2 study evaluating the maximum tolerated dose of MRgFUS in subjects with diffuse intrinsic pontine glioma receiving SDT therapy using 5-ALA is being conducted (NCT05123534). 

## 7. Sensitization Strategies: Radiosensitization

The GBM microenvironment typically features an inadequate vascular network that contributes to tumor hypoxia [153,154]. The hypoxic tumor niche promotes resistance to RT by reducing the supply of oxygen used to generate ROS that damage DNA following radiation [45,155]. Hypoxia also stimulates tumor invasion and metastasis [156]. Given the well-characterized hypoxic microenvironment in GBM, radiosensitization is of interest to increase the efficacy of RT (6). Both thermal and non-thermal effects from FUS can increase tissue oxygenation, rendering FUS an emerging strategy for radiosensitization [157]. FUS-induced cavitation improves the permeability of the BBB and can also cause sonoporation, or temporary small pores in cell membranes that allow for greater oxygen delivery and perfusion [158,159].

Preclinical studies have established the radiosensitizing properties of ultrasound for a variety of malignancies, including breast cancer, bladder cancer, colorectal carcinoma, prostate cancer, melanoma, and fibrosarcoma, among others [160,161,162,163,164,165]. Several studies in GBM and other gliomas have also established the efficacy of FUS prior to RT. Peng et al. illustrated that the combination of RT and FUS increased tumor cell death and inhibited glioma progression in a mouse model of GBM. In addition, the treatment disrupted mechanisms of DNA repair by downregulating repair and checkpoint proteins, such as checkpoint kinase 1 and p53 [166]. Additionally, He et al. found that the combination therapy applied to mice with orthotopic GBM caused autophagosome accumulation and decreased tumor cell viability [167]. 

The application of FUS with the sonosensitizer 5-ALA and a radioenhancer gold/silica nanoparticle allowed Chiang et al. to achieve precision RT using a smaller radiation dose than typical, although the treatments were applied to a human GBM cell line [168]. The 5-ALA and radioenhancer produced ROS and DNA damage after accumulating in tumor cells, sensitizing cells to RT. FUS preceded RT, further increasing radiosensitization. In a separate study, GBM cells exposed to FUS and single-dose irradiation showed significantly reduced metabolic activity and increased apoptotic activity along with greater amounts of DNA double-strand breaks compared to RT alone [169]. Lately, HIFUS has been studied for hyperthermia-mediated radiosensitization [72]. MRgFUS machines can produce HIFUS and use MR thermometry to noninvasively monitor temperature in real-time [161,170]. However, clinical trials in patients with GBM are lacking, and more studies are needed to evaluate the effectiveness of FUS to improve RT for GBM.

## 8. Histotripsy

A non-thermal FUS technique known as histotripsy can be used to mechanically ablate brain tissue and tumors in a precise location without thermal effects [171]. Histotripsy relies on short duration, high amplitude ultrasound pulses to produce acoustic cavitation in tissues that results in inward erosion at a tissue-liquid interface and liquefaction in dense tissue [172,173,174]. The liquefaction creates acellular debris that is resorbed by the body over a few months [175]. In contrast to histotripsy, earlier thermal techniques such as shockwave therapy and HIFUS produced mechanical damage in larger areas with sparser liquefaction [176,177]. These thermal modalities also suffered from a lack of precise margins and side effects from destruction of healthy tissue. The short nature of histotripsy ultrasound pulses, which typically consists of under three acoustic cycles at less than a 1% duty cycle, limits the areas over which cavitation occurs and allows for precisely targeted ablations without extraneous tissue damage [178,179,180]. Consequently, histotripsy is emerging as a popular alternative to thermal ablation and hyperthermia.

Histotripsy ultrasound pulses induce the formation of dense cavitation “bubble clouds” at the focal zone [172,181]. The formation of the “bubble cloud” produces mechanical shearing forces and stress and strain in the target tissue that results in disintegration of cells into an acellular homogenate and fragmentation of the extracellular matrix [182]. Cavitation migration is prevented because the amplitude outside the focal region is insufficient to support dense bubble cloud formation and cavitation [183]. A study in the porcine brain delivered histotripsy pulses to ablate cortical tissue, producing lesions of arbitrary shape and size with dimensions up to 1 cm and well-demarcated, clear margins. Lesions targeting the gyri did not cause significant hemorrhage, edema, or other complications [171]. Since cancer cells have less mechanical stiffness compared to normal tissue, they are generally more vulnerable to the effects of non-thermal ultrasound histotripsy [184,185].

The acellular debris created by histotripsy-induced liquefaction often contains tumor antigens, damage-associated molecular patterns, and heat shock proteins that can recruit a tumor-specific cytotoxic T-cell response [186]. Macrophages and B-cell lymphocytes may also be involved in the inflammatory response stimulated by histotripsy, as both cell populations have been reported to increase in lymphatic tissue following histotripsy [172]. Qu et al. studied mice with melanoma or hepatocellular carcinoma tumors, finding that histotripsy not only stimulated local tumor infiltration by immune cells, but also promoted inflammation at tumor sites not targeted by histotripsy [187]. Notably, this study was not conducted in GBM, and it is unclear whether a similar inflammatory response is observed in the intracranial space.

Histotripsy has been studied in several cancers, including liver, kidney, and prostate cancer [188,189,190,191,192,193,194]. Comparatively fewer studies have investigated histotripsy for brain tumors, although animal studies have confirmed that histotripsy can indeed generate lesions in the brain [171,195]. Continued investigation is necessary to characterize the effectiveness of histotripsy for GBM.

## 9. Liquid Biopsy

Neoplasms of the CNS are traditionally detected on imaging and a definitive diagnosis is conferred only through histological analysis of specimens from surgical resection or biopsy. Surgical resection is generally performed for treatment of GBM, allowing collection of tissue specimen at the time of surgery. However, some patients are not surgical candidates, due to frailty, comorbidities, age, or personal preference. Surgical resections are invasive procedures associated with substantial morbidity, including hemorrhage, infection, and neurological damage [196,197]. Moreover, research into noninvasive treatment options for GBM and other brain tumors may eventually reduce the need for invasive surgery, but pathological diagnosis will still be required to determine the optimal treatment plan. Consequently, there is significant interest in non-invasive approaches, such as blood-based liquid biopsies, for diagnosis of brain tumors. Liquid biopsies ameliorate the risks of invasive surgeries by detecting circulating biomarkers and tumor-derived components [198]. These biopsies are acquired from peripheral blood and detect cell-free DNA (cfDNA), or short DNA fragments typically 180–200 base pairs in length. The DNA fragments accumulate due to rapid tumor growth and turnover that results in the rapid production of circulating tumor DNA [199]. The technology uses polymerase chain reaction and next-generation sequencing technology and allows for early detection of tumors, non-invasive diagnosis of tumors, and monitoring of treatment [199,200]. 

**Figure 3:**
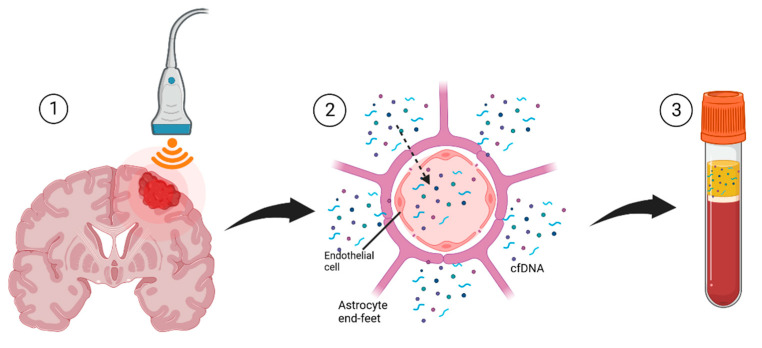
Depiction of the steps for FUS liquid biopsy. (**1**) FUS is used to target the brain tumor in a precise region of interest; (**2**) Oscillation of microbubbles produces acoustic cavitations that cause transient opening of the BBB and release of biomarkers across the endothelial cells lining the BBB; (**3**) The biomarkers diffuse into the peripheral circulation and are collected in a blood draw for analysis. (**3**). Created with BioRender.com.

Liquid biopsies have been studied in several types of cancers with varying success, with circulating tumor DNA detectable in pancreatic, breast, colorectal, ovarian, and other cancers [201]. However, its application in brain tumors has been limited by the impermeability of the BBB, which often prevents tumor biomarkers from reaching the peripheral circulation [202]. Indeed, fewer than 10% of patients with gliomas have detectable cfDNA. In contrast, investigations into other solid tumors, such as pancreatic, ovarian, colorectal, bladder, gastroesophageal, and breast cancers, have reported finding cfDNA in most patients [197,201]. As a result, researchers have begun to study ways to non-invasively improve the detection of biomarkers in peripheral circulation. By transiently opening the BBB to allow for the diffusion of tumor DNA, FUS represents one such approach and has emerged as a promising method for liquid biopsies of brain tumors given its non-invasiveness and precision in spatial localization (Figure 3) [202].

### Focused Ultrasound for Liquid Biopsies

FUS for improved biomarker release has been studied in several animal models of GBM (Table 2). Zhu et al. tested two GBM tumor models in which mice were injected with enhanced green fluorescent protein-transduced GBM cells [196]. FUS was applied to generate oscillation of microbubbles and disruption of the BBB. Four minutes after FUS treatment, blood was collected, and quantitative polymerase chain reaction performed to detect the fluorescent protein. Circulating levels of fluorescent protein mRNA were over 1500-fold higher in the mice that underwent FUS-mediated BBB disruption. Different acoustic pressures were tested using a MRgFUS machine, with the researchers finding that lower pressures resulted in more mRNA release compared to higher pressures. Additionally, higher pressure led to increased hemorrhage [196]. In a subsequent study, the research team used lower acoustic pressures to avoid hemorrhage in the brain, confirming that acoustic pressures of 0.59 MPa were sufficient for detection of fluorescent protein mRNA in a mouse GBM model using liquid biopsy. These low acoustic pressures resulted in significantly less microhemorrhage compared to higher acoustic pressures tested [203].

Additionally, Pacia et al. tested FUS for liquid biopsy in a porcine model, finding significant increases in plasma concentration of brain-specific biomarkers after FUS sonication. Tissue damage was not detected on MRI or histology. However, a specific tumor model was not investigated [198]. These studies continue to help elucidate the effectiveness and utility of FUS liquid biopsy, while also allowing for FUS parameter optimization. 

Clinical studies investigating FUS for enhanced liquid biopsy of brain tumors are limited, but one study by Meng et al. suggested that FUS can enhance circulating tumor biomarker detection by increasing permeability of the BBB [202]. In a study of nine patients with GBMs, MRgFUS was used to study biomarkers in blood samples collected before and after sonication. They found that MRgFUS enhanced plasma cfDNA collected a half-hour after sonication. DNA methylation profiling of the cfDNA suggested a cancer signature unique to the patient. A larger study is now being initiated that will involve 50 patients undergoing partial tumor ablation with MRgFUS or traditional tumor biopsy and excision with the subsequent collection of blood and cerebrospinal fluid (NCT04940507) [205]. 

FUS-enabled liquid biopsy has major benefits over surgical biopsy for purposes of diagnosis. Following diagnosis, FUS could be used to improve BBB permeability and allow improved tumor targeting by chemotherapeutic agents, ablate tumor tissue with HIFUS-mediated hyperthermia, and sensitize tissue to radiation, amongst other modalities. Additional studies are needed to further evaluate potential risks of FUS for liquid biopsies, including whether the increased permeability of the BBB to tumor components could induce metastatic spread. Extracranial metastasis of GBM is rare, but this may in part reflect the short survival time and the difficulty tumor tissue faces spreading across the BBB [196]. Liquid biopsies will improve the development of personalized treatment plans by detecting resistant or sensitive tumor variants as well as monitoring a patient’s response to specific treatments [197,202]. Ultimately, FUS has improved the feasibility of liquid biopsies for GBM, which may lead to improvements in detection and treatment for patients. 

## 10. Challenges and Opportunities

Research into applications of FUS for GBM has progressed at a rapid pace in recent years, spurred by technological advancements and promising preclinical results. Clinical trials using FUS for GBM have obtained approval by the Food and Drug Administration, but the technology is not yet approved for routine clinical use. A favorable safety profile has been demonstrated, particularly with LIFUS. The transient nature of BBB opening using LIFUS, along with the precise delivery of FUS beams using MRgFUS machines, limit side effects and systemic toxicity [206]. Anastasiadis et al. illustrated the safety of MRgFUS for BBB opening in a Phase 0 clinical trial of four patients with infiltrating gliomas, which successfully opened the BBB without evidence of MRI or microscopic tissue injury [65]. Similarly, a Phase 1 study of five patients with high-grade gliomas showed that MRgFUS could produce BBB opening without clinical or radiographic adverse events [207]. However, larger sample sizes will be needed with long-term follow-up to validate safety. FUS-mediated hyperthermia, although used for treatment of other cancers, has been limited in GBM by potential side effects, including skin burns and ablation of healthy tissue [208].

A limitation of the transient nature of FUS-mediated BBB opening is that repeat treatments are needed to permit multiple administrations of therapeutic agents. In contrast, other FUS modalities, such as thermal ablation and histotripsy, may require fewer treatments. Intracranial FUS implants, such as the SonoCloud, could also be used to reduce follow-up treatment visits. Improvements in microbubble technology and longer microbubble half-life can also increase the treatment window for drug delivery after FUS [209]. 

Investigation into the optimal FUS modalities for GBM treatment is ongoing. Given the aggressive nature of GBM, the greatest improvements in patient survival may stem from treatments that combine different FUS modalities, such as tumor ablation and BBB opening, or those that combine FUS with alternative treatment options, such as local drug delivery. Additionally, although extensive research has been conducted for intracranial neoplasms, investigation of FUS for tumors of the spine or spinal cord are lacking. GBM is not limited to the brain, and can cause significant morbidity and mortality when it arises within the spinal cord [210]. These intramedullary lesions are similarly associated with short overall survival, and complete surgical resection is usually unachievable due to the infiltrating nature of the tumor and high risk of neurological deficits from resection of healthy cord tissue [211]. FUS technology will need to account for differences in the thickness and irregularity of the bony spine compared to the calvarium, and avoiding healthy tissue will be critical for reducing iatrogenic deficits. MRgFUS has been studied for treatment of low back pain caused by facet joint osteoarthritis and for treatment of bone metastases, and extension of treatment to the spine may be forthcoming [208,212].

## 11. Conclusions

The mainstay of treatment for GBM consists of surgical resection, TMZ chemotherapy, and RT, but patients face a poor prognosis and short overall survival time. Transcranial FUS, applied under MR guidance, is emerging as a new technology for treatment of intracranial pathologies, including GBM. MRgFUS can transiently open the BBB, allowing improved penetration of drugs and chemotherapy to the brain. The technology is precise and minimizes side effects to the surrounding tissue. Nanoparticles can be used to localize compounds to the tumor site following FUS treatment. Other applications of FUS for GBM include tumor ablation using HIFUS-mediated hyperthermia, stimulation of the immune system to target tumor cells, and administration of sonosensitizers that produce ROS and contribute to tumor apoptosis in the presence of FUS. Additionally, FUS is being examined for its radiosensitizing properties and its ability to produce liquefactive necrosis without thermal effects. An increasing number of clinical trials examining FUS for GBM and other brain tumors have been conducted in recent years and have illustrated its feasibility and favorable safety profile, although more studies are needed to validate efficacy. FUS technology will continue to undergo improvements and refinements in the future and may one day play a critical role in the treatment paradigm for GBM, extending patient survival and improving quality of life in patients with GBM.

## Figures and Tables

**Table 1:** Preclinical investigations into SDT for treatment of GBM.

Sonosensitizer	Application/Additional Treatment	Cell Line	Intensity (W/cm^2^)	Frequency (MHz)	Exposure Time (min)	Main Findings	Ref.
5-ALA	Standard SDT	C6/U87	10	1.1	3	Reduction in tumor cell size and viability	[101]
5-ALA	Standard SDT	C6	5.5	1.06	20	Inhibition of tumor growth, significantly improved survival.	[115]
5-ALA	Standard SDT	F98	20	0.22	4	Reduced tumor cell viability, induction of apoptosis, suppression of tumor proliferation and invasion, minimal damage to normal brain tissue.	[114]
5-ALA	Standard SDT	U87/U251	2	3	3	Inhibition of tumor cell growth, increased apoptotic death, prolonged survival.	[111]
5-ALA	Standard SDT	RG2	2–6	1	3	Decreased cell viability, increased chromatin condensation and apoptosis.	[119]
5-ALA	Standard SDT	C6	0.33–8	1.06	—	Average threshold intensity causing tumor cell death determined as 5.7 W/cm^2^.	[120]
5-ALA/PPIX	Standard SDT	C6/U87	0.16	1	1	Enhanced tumor cell cytotoxicity and increased induction of apoptosis.	[118]
DVDMS	Standard SDT	U87	0.32	0.97	3	Significant cytotoxicity	[121]
DVDMS	Standard SDT	U373	0.45	1	1	Significant loss of tumor cell viability and increased apoptosis, caspase-3, and DNA fragmentation.	[122]
Fluorescein	Standard SDT	C6	2–6	0.35	20	Significant inhibition of ectopic glioma outgrowth.	[123]
HMME	Standard SDT	C6	0.5	1	2	Inhibition of tumor growth and angiogenesis, induction of apoptosis.	[117]
HMME	Standard SDT	C6	1	0.5	1	Increased induction of apoptosis, ROS production, and cyt-c along with decreased MMP.	[124]
HMME	Standard SDT	C6	1	0.5	1	Apoptosis, ROS production, decreased MMP, and release of cytochrome c.	[125]
Nanoparticles							
Ce6	Fe_3_O_4_ + Ce6 NPs	C6	1	1	1	Significant inhibition of tumor growth, prolonged median survival, no adverse effects on healthy tissues	[126]
Ce6	Ce6 + HCQ liposomal NPs	GL261	1	1	1	Significant inhibition of tumor growth, prolonged survival time	[127]
Ce6	Mn^2+^-chelated Ce6 NPs	U87	1	0.8	—	Complete suppression of subcutaneous tumor growth and delayed progression of orthotopic tumor growth.	[128]
DVDMS	DVDMS Liposomal NPs	C6	1	1	1	Suppression of tumor growth, increased median survival time and good biocompatibility	[116]
DVDMS	Mn^2^-chelated DVDMS NPs	U87	0.5	0.5	5	Inhibition of tumor growth.	[129]
Indocyanine green	Silica NPs loaded with indocyanine green	U87	1.5	1	5	Significant inhibition of tumor growth, increased median survival	[130]
IR780	Angiopep-2 + PLGA + IR780 + MnO_2_ NPs	U87	1	1	1	Improved targeting and deeper penetration into tumors, significant inhibition of tumor growth and distal metastasis, lack of systemic toxicity.	[131]
IR780	IR780 NPs	U87	0.2–0.4	1	3	Significant inhibition of tumor growth, induction of apoptosis in tumors, no obvious toxicity.	[132]
HMME	YVO_4_:Nd^3+^-HMME NPs with MnO_2_ shell	C6	0.7	3	4	Inhibition of tumor growth	[133]
Hypocrellin	PEG-PGLA NPs with hypocrellin	U87	0.8	1	5	Slower tumor growth rates	[134]
PPIX	MnO^2^—transferrin NPs loaded with PPIX	C6	1.5	1	3	Suppression of tumor growth, favorable biocompatibility, and safety.	[135]
Additional therapies							
5-ALA	Combined with hyperthermotherapy	SNB19/U87	1–2	1	2	Significant reduction in tumor cell viability, increased apoptosis induction	[100]
5-ALA	Combined with celecoxib	Mouse glioma cells	2	1	2	Decreased tumor volume, improved survival	[136]
DVDMS	Combined with PDT	U118/U87	0.5	1	1–3	Inhibition of glioma cell proliferation, induction of tumor cell apoptosis	[113]
HMME	Combined with Ca^2+^ channel antagonist	U87	0.5	0.04	1	Tumor volume significantly suppressed.	[137]
HMME	Combined with PDT	C6	0.5	1	1.5	Significantly higher tumor growth inhibition rate, apoptosis rate ROS generation.	[138]
TiO_2_	Combined with anti-EGFR antibody	U87/U87de2–7	1	1	1	Reduced tumor cell viability	[139]

5-ALA—5-Aminolevulinic acid; Ce6—chlorin e6; DVDMS—sinoporphyrin sodium; EGFR—epidermal growth factor receptor; HCQ—hydroxychloroquine; HMME—hematoporphyrin monomethyl ether; NP—nanoparticle; PEG—polyethylene glycol; PGLA—poly (lactic-co-glycolic acid); PPIX—protoporphyrin IX; SDT—sonodynamic therapy.

**Table 2:** Ultrasound parameters tested in animal and clinical studies of FUS liquid biopsies.

Transducer	Transducer Focus	Acoustic Pressure	Duty Cycle	Pulse Repetition Frequency	Exposure Duration	Refs.
Animal Studies						
VIFU 2000; Alpinion US Inc., Bothell, WA, USA	1.5 MHz	3.82 MPa	1%	1 Hz	2 min	[196]
Sonalleve V2, Profound Medical Inc., Mississauga, ON, Canada	1.44 MHz	1.48 MPa 2.74 MPa 3.53 MPa	1%	1 Hz	2 min	
Sonalleve V2, Profound Medical Inc., Mississauga, ON, Canada	1.44 MHz	0.59 MPa, 1.29 MPa, 1.58 MPa	1%	1 Hz	4 min	[203]
Imasonics, Voray sur l’Ognon, France	650 kHz	1.5 MPa	1%	1 Hz	3 min	[198]
Human Studies						
ExAblate Neuro hemispheric device (InSightec, Tirat Carmel, Israel)	220 kHz	500 kPa	0.74%	33 Hz	50 s	[202,204]

min—minutes, MPa—Megapascal, s—seconds.

## Abbreviations

5-ALA	5-Aminolevulinic acid
BBB	Blood–brain barrier
cfDNA	Cell-free DNA
CNS	Central nervous system
FUS	Focused ultrasound
GBM	Glioblastoma multiforme
MRI	Magnetic resonance imaging
MRgFUS	Magnetic resonance guided focused ultrasound
RT	Radiation therapy
SDT	Sonodynamic therapy
TMZ	Temozolomide
